# Impact of *BRCA* Mutation Status on Tumor Dissemination Pattern, Surgical Outcome and Patient Survival in Primary and Recurrent High-Grade Serous Ovarian Cancer: A Multicenter Retrospective Study by the Ovarian Cancer Therapy-Innovative Models Prolong Survival (OCTIPS) Consortium

**DOI:** 10.1245/s10434-022-12459-3

**Published:** 2022-09-09

**Authors:** Jacek Glajzer, Dan Cacsire Castillo-Tong, Rolf Richter, Ignace Vergote, Hagen Kulbe, Adriaan Vanderstichele, Ilary Ruscito, Fabian Trillsch, Alexander Mustea, Caroline Kreuzinger, Charlie Gourley, Hani Gabra, Eliane T. Taube, Oliver Dorigo, David Horst, Carlotta Keunecke, Joanna Baum, Timothy Angelotti, Jalid Sehouli, Elena Ioana Braicu

**Affiliations:** 1 Department of Gynecology, Charité–Universitätsmedizin Berlin, Corporate Member of Freie Universität Berlin and Humboldt Universität zu Berlin, Augustenburger Platz 1, Berlin, Germany; 2 Tumorbank Ovarian Cancer Network, Berlin, Germany; 3 Department of Obstetrics and Gynecology, Comprehensive Cancer Center, Medical University of Vienna, Waehringer Guertel 18-20, Vienna, Austria; 4 Division of Gynecological Oncology, Department of Gynaecology and Obstetrics, Leuven Cancer Institute, Universitaire Ziekenhuizen Leuven, Katholieke Universiteit Leuven, Herestraat 49, Leuven, Belgium; 5 Gynecology Division, Department of Medical and Surgical Sciences and Translational Medicine, Sant’Andrea University Hospital, Sapienza University of Rome, Via di Grottarossa 1035, Rome, Italy; 6 Department of Obstetrics and Gynecology, University Hospital LMU Munich, Munich, Germany; 7 Department of Gynecology and Gynecological Oncology, University Hospital Bonn, Venusberg-Campus 1, Bonn, Germany; 8 Institute of Science and Technology Austria, Am Campus 1, Klosterneuburg, Austria; 9 Nicola Murray Centre for Ovarian Cancer Research, University of Edinburgh Cancer Research UK Centre, MRC Institute of Genetics and Cancer,, Western General Hospital, Crewe Road South, Edinburgh, UK; 10 Ovarian Cancer Action Research Centre, Department of Surgery and Cancer, Imperial College London, London, UK; 11 Institute of Pathology, Charité-Universitätsmedizin Berlin, Campus Mitte, Berlin, Germany; 12 Department of Obstetrics and Gynecology, Division of Gynecologic Oncology, Stanford University School of Medicine, Stanford, CA USA; 13 Department of Anesthesiology, Perioperative and Pain Medicine, 300 Pasteur Drive H3580, Stanford, CA USA; 14 Department of Oral and Cranio-Maxillofacial Surgery, University Hospital Erlangen, Glückstraße 11, Erlangen, Germany

## Abstract

**Background:**

This study seeks to evaluate the impact of breast cancer (*BRCA*) gene status on tumor dissemination pattern, surgical outcome and survival in a multicenter cohort of paired primary ovarian cancer (pOC) and recurrent ovarian cancer (rOC).

**Patients and Methods:**

Medical records and follow-up data from 190 patients were gathered retrospectively. All patients had surgery at pOC and at least one further rOC surgery at four European high-volume centers. Patients were divided into one cohort with confirmed mutation for *BRCA1* and/or *BRCA2* (*BRCA*mut) and a second cohort with *BRCA* wild type or unknown (*BRCA*wt). Patterns of tumor presentation, surgical outcome and survival data were analyzed between the two groups.

**Results:**

Patients with *BRCA*mut disease were on average 4 years younger and had significantly more tumor involvement upon diagnosis. Patients with *BRCA*mut disease showed higher debulking rates at all stages. Multivariate analysis showed that only patient age had significant predictive value for complete tumor resection in pOC. At rOC, however, only *BRCA*mut status significantly correlated with optimal debulking. Patients with *BRCA*mut disease showed significantly prolonged overall survival (OS) by 24.3 months. Progression-free survival (PFS) was prolonged in the *BRCA*mut group at all stages as well, reaching statistical significance during recurrence.

**Conclusions:**

Patients with *BRCA*mut disease showed a more aggressive course of disease with earlier onset and more extensive tumor dissemination at pOC. However, surgical outcome and OS were significantly better in patients with *BRCA*mut disease compared with patients with *BRCA*wt disease. We therefore propose to consider *BRCA*mut status in regard to patient selection for cytoreductive surgery, especially in rOC.

Maximum tumor reduction and platinum-based chemotherapy eventually followed by maintenance with poly(ADP-ribose) polymerase inhibitors (PARPi) alone or in combination with antiangiogenesis is the standard treatment for pOC.^1^ However, in the recurrent state of disease, selection of the best therapeutic option depends on the recurrence timing and pattern and is considerably more complex.^2^ Recently published data of a randomized phase III trial conducted by Harter et al. demonstrated that secondary cytoreduction significantly extends OS as well as PFS if complete tumor resection is achieved.^3^ Currently, the Arbeitsgemeinschaft Gynaekologische Onkologie (AGO) score, consisting of three items (complete resection at initial surgery, good performance status, and absence of ascites) represents a well-validated tool to identify patients most likely to achieve complete secondary cytoreduction.^4–6^ However, studies have shown that patients with negative AGO score can still achieve complete resection, thus further refinement of the score is necessary.^7–9^ Hence, additional individual criteria to inform the selection of patients suitable for secondary debulking surgery must be identified.^10^

Since ovarian cancer is known to show high interindividual heterogeneity in clinical presentation and treatment response, as well as high recurrence risk, our main focus in recent research has been to determine the impact of *BRCA* status on this diversity.^11^ In this context, *BRCA* mutation status predicts the effects of PARPi with impressive clinical results.^12^ Moreover, the effectiveness of PARPi as maintenance treatment for newly diagnosed advanced ovarian cancer has also been proven.^13^ Nevertheless, surgical cytoreduction will continue to be an important therapeutic strategy to efficiently remove a large number of cancer cells at a time, even in the situation of recurrence.^14^ Patients with no residual tumor mass following primary tumor debulking were the ones who benefited most from maintenance with PARPi.^15^ However, the relevance of *BRCA* mutation status as a predictive factor for platinum sensitivity and targeting point for individualized therapy options is increasing.^16,17^ With the increasing importance of surgical treatment in rOC, the role of *BRCA* mutation status in secondary resection has not yet been sufficiently investigated.^18^

It is the aim of this multicenter retrospective study to evaluate the impact of *BRCA* mutation status on clinical presentation and surgical outcome in the primary and recurrent disease state, as well as patient survival.

## Patients and Methods

### Patient Enrollment

Paired tumor tissue samples, surgical reports, medical records and follow-up data of patients from both pOC and all available rOC treatment lines were gathered retrospectively from the Ovarian Cancer Therapy-Innovative Models Prolong Survival (OCTIPS, agreement no. 279113-2) consortium database. All patients underwent cytoreductive surgery for pOC and rOC disease with maximum effort followed by platinum-based chemotherapy. Tissue samples irrespective of tumor stage were harvested during tumor debulking surgery between 1984 and 2019 and subsequently underwent histopathological analysis to confirm high-grade serous ovarian cancer (HGSOC) diagnosis. Tumor tissue samples were also used to retrospectively determine *BRCA* mutation status. After completion of surgery, a standardized interview with the surgeon was conducted to assess the macroscopic spread of tumor mass and the surgical procedures performed. If necessary, surgical reports were additionally reviewed to complement surgical data. All patients were treated in one of the following accredited European gynecological oncology referral centers: Charité-Universitätsmedizin Berlin (Germany), Medical University of Innsbruck (Austria), Katholieke Universiteit Leuven (Belgium) and University of Edinburgh (United Kingdom). The study received favorable approval from each local ethics committee (EK207/2003, EK260, ML2524, and EK130113).

Inclusion criteria were presence of HGSOC with availability of thorough clinicopathological and complete surgical documentation for both primary disease and at least one recurrence. Exclusion criteria were lack of the aforementioned data, other histologies, or inadequate follow-up.

### Patient Clinicopathological Data

Informed consent was collected as per local ethics committee requirements prior to enrollment into the study. Clinicopathological data were obtained from the OCTIPS consortium database and processed anonymously. The 1988 International Federation of Gynecology and Obstetrics (FIGO) classification was used to determine clinical stage and the 2014 World Health Organization (WHO) classification to histologically assess the tumor tissue samples.^19^ To evaluate the patterns of intraoperative disease presentation, the abdominal cavity was divided into three compartments: lower abdomen (level 1, including uterus, Douglas, bladder and ureters, rectum, sigma, and vaginal stump), periumbilical section (level 2, including colon and small intestine), and upper abdomen (level 3, including omentum majus, bursa omentalis, spleen, stomach, liver, and diaphragm), according to the concept of the Intraoperative Mapping of Ovarian cancer (IMO) system.^20^ Extension of surgical procedures was measured by application of the surgical complexity scoring (SCS) system, defined elsewhere.^21^ OC recurrence was defined on the basis of clinical examination, cross-sectional imaging using the response evaluation criteria in solid tumors (RECIST) criteria, and serum marker evaluation.^22^ Residual tumor was defined as negative by the surgeon if macroscopically absent at the end of cytoreduction.

### Statistical Analysis

IBM SPSS version 24 (IBM Corporation, Armonk, NY) was used to perform the statistical analysis of the patient cohort at Charité-Universitätsmedizin Berlin. Differences between the *BRCA*mut and *BRCA*wt cohorts concerning the investigated factors were analyzed using Pearson’s chi-squared test, Fisher’s exact test, Mann–Whitney *U* test, and Kendall’s tau–*b* test where appropriate.

OS was defined as the period from first diagnosis of ovarian cancer until death or last contact. PFS was determined as the interval from surgery until progression of disease or death. Both OS and PFS were estimated by Kaplan–Meier analysis, and survival differences were analyzed using the log-rank test. Significance level was set at a two-sided *p*-value ≤ 0.05. Multivariate analysis was performed to estimate the probability of complete tumor resection during pOC and rOC surgery, consisting of *BRCA* mutation status, patient age, and SCS.

## Results

### Patient Characteristics

**Fig. 1:**
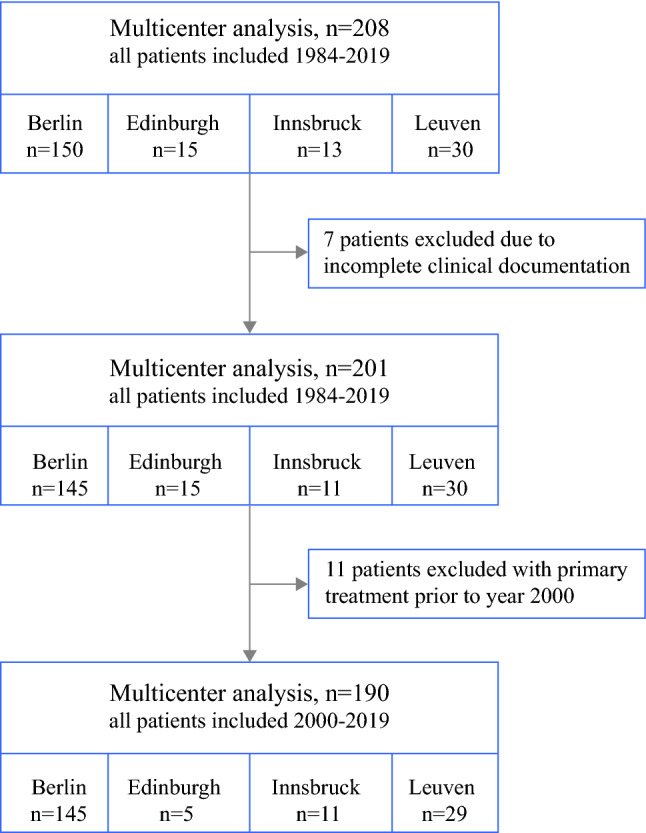
Study flow diagram

A total of 208 patients were included in the study. To ensure that all patients were treated with surgery followed by platinum-based chemotherapy, all cases prior to 2000 were excluded. Seven further patients had to be excluded from the study due to insufficient clinicopathological documentation. In total, 190 patients were included in the final analysis of the study. Patient enrollment is visualized in Fig. 1. Mean follow-up from primary surgery was 51.7 months (range 6.7–214 months), with 124 deaths occurring during that period. All included patients had HGSOC. The majority of diagnoses (77.89%) were FIGO stage 3. A total of 138 women (72.6%) had known *BRCA* status [30 (15.8%) *BRCA1*mut, 19 (10%) *BRCA2*mut, and 92 (48.4%) *BRCA*wt] while it was unknown in 52 (27.4%) at the end of follow-up. Patients with a known *BRCA* mutation were on average 4 years younger at time of diagnosis (53 versus 57 years, *p* = 0.018). Over 90% of all patients were Caucasian, thus no further specification of ethnicity was performed. Patient characteristics are presented in Table 1.

**Table 1:** Patient characteristics

	All patients	*BRCA*mut	*BRCA*wt/unknown	*p*-value
*Whole cohort, n (%)*	190 (100)	46 (24.2)	92 (48.4)/52 (27.4)	
*Center, n (%)*				
Berlin	145	37	108	
Innsbruck	11	2	9	
Leuven	29	6	23	
Edinburgh	5	1	4	
*Median age, years (range)*	55 (20–75)	53 (37–69)	57 (20–75)	**0.018**
*Final diagnosis, n (%)*				0.220
Ovarian cancer	182 (95.8)	42 (91.3)	140 (97.2)	
Fallopian tube carcinoma	4 (2.1)	2 (4.3)	2 (1.4)	
Primary peritoneal carcinoma	4 (2.1)	2 (4.3)	2 (1.4)	
*Histologic type at first diagnosis, n (%)*				0.589
Serous	179 (94.2)	46 (100)	133 (92.4)	
Endometrioid	5 (2.6)	0 (0)	5 (3.5)	
Other	6 (3.16)	0 (0)	6 (4.17)	
*Grading at first diagnosis, n (%)*				0.760
I	4 (2.1)	1 (0.5)	3 (2.1)	
II	36 (18.9)	7 (15.2)	29 (20.1)	
III	150 (78.9)	38 (82.6)	112 (77.8)	
*FIGO stage, n (%)*				0.610
I	3 (1.6)	0 (0)	3 (2.1)	
II	10 (5.3)	2 (4.3)	8 (5.6)	
III	148 (77.9)	37 (80.4)	111 (77.6)	
IV	29 (15.3)	7 (15.2)	22 (15.3)	
*Primary treatment strategy*				0.673
Primary debulking surgery	141 (74.2)	36 (78.3)	105 (72.9)	
Interval debulking surgery	16 (8.4)	4 (8.7)	12 (8.3)	
Completion surgery	33 (17.4)	6 (13)	27 (18.8)	

Bold numbers represent statistically significant p-values (*p* ≤ 0.05). Percentage numbers are presented in relation to patients with available information

### Tumor Dissemination Pattern and Surgical Outcome

No statistical significance was found between the two groups in terms of SCS based upon the complexity and number of procedures performed during primary and secondary cytoreductive surgery. In particular, intermediate surgical complexity was achieved in both groups during pOC, consisting of 6.0 points for the *BRCA*mut group (16.2% low, 62.2% intermediate, and 21.6% high surgical complexity) and 6.0 points for the *BRCA*wt cohort (10.2% low, 62% intermediate, and 27.8% high; *p* = 0.407). During secondary debulking, the median surgical complexity score was lower compared with primary surgery, with 3.0 points for the *BRCA*mut group (70% low and 30% intermediate) and 2.0 points for the *BRCA*wt group (79.5% low and 20.5% intermediate; *p* = 0.089). Patient characteristics are presented in Table 1.

Results for surgical outcome in relation to *BRCA* status are presented in Table 2. Patients with *BRCA*mut disease showed higher disease burden at IMO level 2 during primary surgery compared with patients with *BRCA*wt disease (91.7% versus 75%, *p* = 0.034). No other significant differences could be identified between the two groups in terms of tumor dissemination at primary or recurrent disease stages.

**Table 2:** Surgical outcome

	Primary			1st recurrence			2nd/3rd recurrence		
	*BRCA*mut	*BRCA*wt/unknown	*p*-Value	*BRCA*mut	*BRCA*wt/unknown	*p*-Value	*BRCA*mut	*BRCA*wt/unknown	*p*-Value
*Intraoperative ascites, n (%)*			0.152			0.522			0.910
No ascites	18 (39.1)	77 (53.5)		31 (79.5 )	87 (74.4)		9 (75)	33 (80.5)	
< 500 ml	11 (23.9)	33 (22.9)		7 (17.9)	21 (17.9)		2 (16.7)	5 (12.2)	
> 500 ml	17 (37)	34 (23.6)		1 (2.6)	9 (7.7)		1 (8.3)	3 (7.3)	
*Median preoperative serum CA-125 level, U/ml (range)*	731 (7–9000)	199 (7–23700)	**0.021**	241 (21–3936)	96 (7–21591)	**0.006**	210 (0–7286)	156 (0–12250)	0.749
*Tumor dissemination pattern, n (%)*									
IMO**-**level 1 (lower abdomen)	33 (91.7)	101 (93.5)	0.711	20 (64.5)	59 (74.7)	0.347	8 (72.7)	33 (86.8)	0.355
IMO- level 2 (middle abdomen)	33 (91.7)	81 (75)	**0.034**	20 (64.5)	63 (79.7)	0.138	7 (63.6)	25 (65.8)	0.895
IMO-level 3 (upper abdomen)	24 (66.7)	61 (56.5)	0.331	21 (67.7)	52 (65.8)	0.848	7 (63.6)	14 (36.8)	0.169
*Median surgical complexity score (range)*	6.0 (1-11)	6.0 (0-12)	0.407	3.0 (0-6)	2.0 (0-5)	0.089			
*Postoperative tumor residual, n (%)*			0.329			**0.004**			**0.005**
No macroscopic residual tumor	38 (82.6)	109 (75.7)		32 (82.1)	66 (56.4)		9 (75)	11 (26.8)	
Macroscopic residual tumor	8 (17.4)	35 (24.3)		7 (17.9)	51 (43.6)		3 (25)	30 (73.2)	
*Peritoneal carcinomatosis, n (%)*	35 (76.1)	109 (75.7)	0.957	22 (56.4)	71 (60.7)	0.638	7 (63.6)	28 (68.3)	0.770
*Lymph node involvement, n (%)*			0.127			0.38			0.084
N0	11 (23.9)	32 (22.2)		6 (19.4)	4 (4.8)		0 (0)	4 (10.5)	
N1	18 (39.1)	66 (45.8)		7 (22.6)	17 (20.2)		5 (45.5)	6 (15.8)	
Unknown	17 (37)	46 (31.9)		18 (58.1)	63 (75.0)		6 (54.5)	28 (73.7)	
*Distant metastatic spread, n (%)*			0.889			0.876			0.881
M0	21 (45.7)	72 (50)		18 (60.0)	58 (54.7)		5 (45.5)	20 (54.1)	
M1	7 (15.2)	19 (13.2)		4 (13.3)	16 (15.1)		1 (9.1)	3 (8.1)	
Mx	18 (39.1)	53 (36.8)		8 (26.7)	32 (30.2)		5 (45.5)	14 (37.8)	

Bold numbers represent statistically significant p-values (*p* ≤ 0.05). Percentage numbers are presented in relation to patients with available information

Complete tumor resection was achieved more often in the *BRCA*mut group during primary debulking surgery (82.6% versus 75.7%, *p* = 0.329), as well as the first (82.1% versus 56.4%, *p* = 0.004) and second/third relapse (75% versus 26.8%, *p* = 0.005), reaching statistical significance. Multivariate analysis of surgical outcome at primary debulking surgery revealed that only age was a significant predictor (OR 1.062; 95% CI 1.011–1.116; *p* = 0.016) of complete tumor resection, whereas *BRCA* mutation status (OR 0.663; CI 95% 0.202–2.179; *p* = 0.499) and SCS (OR 0.999; CI 95% 0.193–5.180; *p* = 0.999) showed no statistical significance. During secondary debulking surgery, however, only *BRCA* mutation status remained a statistically significant predictive factor (OR 0.214; CI 95% 0.078–0.587; *p* = 0.003) for complete tumor resection. Age (OR 0.972; CI 95% 0.939–1.007; *p* = 0.115) and surgical complexity (OR 0.557; CI 95% 0.207–1.501; *p* = 0.247) failed to reach statistical significance.

Focusing on the intraoperative presence of peritoneal carcinomatosis, as described by the surgeon, no significant difference could be determined between the *BRCA*mut and *BRCA*wt cohorts. In particular, involvement of the peritoneum was 76.1% versus 75.7% (*p* = 0.957) during pOC, 56.4% versus 60.7% (*p* = 0.638) in first recurrence, and 63.6% versus 68.3% (*p* = 0.770) in second/third recurrence, respectively.

Preoperative cancer antigen 125 (CA-125) levels were significantly higher in the *BRCA*mut cohort of pOC (731 versus 199 U/ml; *p* = 0.021) and first recurrence (241 versus 96 U/ml, *p* = 0.006). No significant difference could be detected during further recurrences.

Intraoperative ascites volumes did not show significant differences at pOC. The majority of patients in both groups had no ascites at initial presentation (53.5% versus 39.1%; *p* = 0.152). No significant differences in intraoperative ascites levels could be measured in the relapse situation.

### Survival

**Fig. 2:**
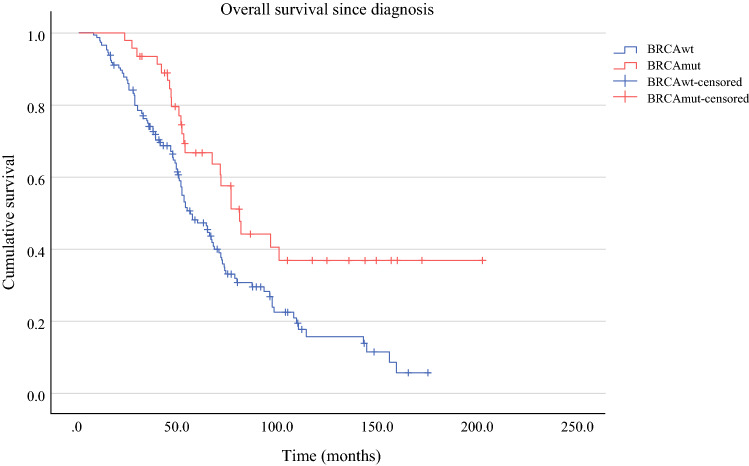
OS from surgery for pOC between the *BRCA*mut and *BRCA*wt cohorts

**Fig. 3:**
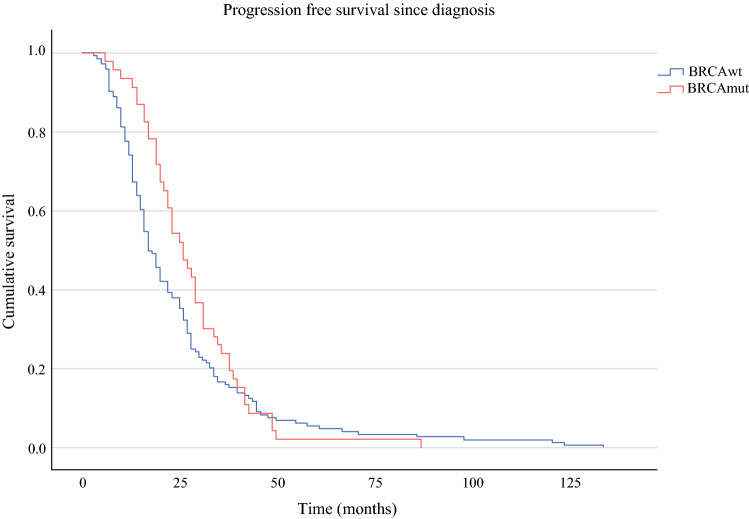
PFS after surgery for pOC between the *BRCA*mut and *BRCA*wt cohort

**Fig. 4:**
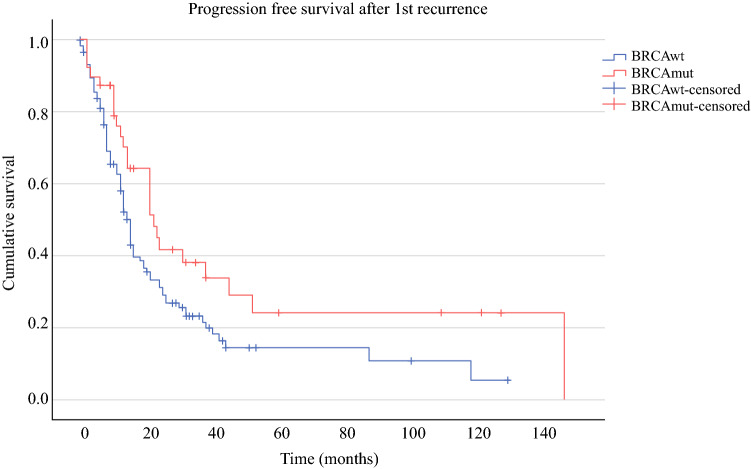
PFS after surgery for first OC recurrence between the *BRCA*mut and *BRCA*wt cohorts

**Fig. 5:**
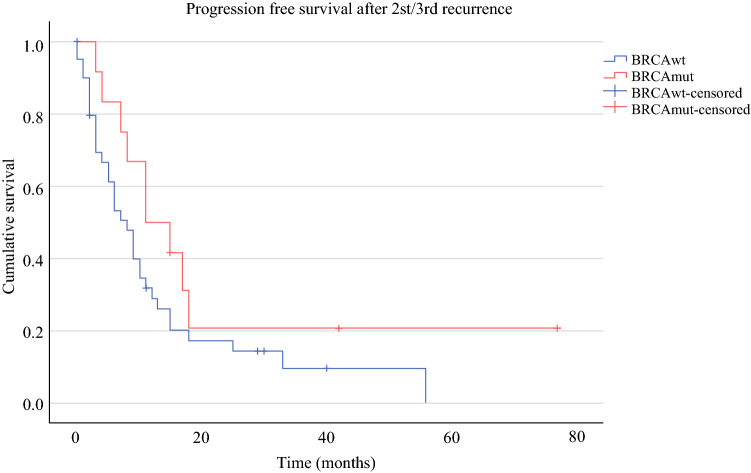
PFS after surgery for second/third OC recurrence between the *BRCA*mut and *BRCA*wt cohorts

OS was significantly prolonged in the *BRCA*mut cohort compared with the *BRCA*wt patients (80.6 versus 56.3 months, *p* = 0.003, Fig. 2). PFS for *BRCA*mut and *BRCA*wt subgroups was 26 and 17 months in the primary situation (*p* = 0.182, Fig. 3), 22 versus 15 months after first relapse (*p* = 0.025, Fig. 4), and 11 versus 8 months after second or third relapse (*p* = 0.118, Fig. 5).

## Discussion

### Summary of Main Findings

This study was conducted to evaluate and compare the role of *BRCA*mut status in clinical presentation, surgical outcome in the primary and recurrent disease state, as well as patient survival in a multicenter cohort of patients with HGSOC disease with paired primary and relapsed tissue samples. The study showed that *BRCA*mut correlates with more frequent extrapelvic presentation at initial diagnosis. Moreover, the *BRCA*mut cohort presented with higher optimal debulking rates at all stages. Patients with *BRCA*mut disease showed a significant median OS benefit of 24.3 months compared with the *BRCA*wt cohort. At secondary debulking surgery, multivariate analysis confirmed positive *BRCA* status as an independent favorable factor for complete macroscopic tumor resection. Patient characteristics of our study cohort were well comparable to other published OC collectives in terms of patient age at primary diagnosis and percentage of *BRCA*mut, for example.^23,24^

### Results in the Context of Published Literature

The comparison of tumor dissemination patterns revealed that patients with *BRCA*mut disease showed a more aggressive course of disease during pOC, with significantly more extensive involvement of the mid-abdomen compared with the *BRCA*wt group. However, this phenomenon vanishes throughout the course of disease and no significant differences could be determined during rOC stages. This was confirmed by Marchetti et al., who examined the tumor dissemination pattern between patients with *BRCA*mut and *BRCA*wt disease and could not detect any significant differences at recurrence.^25^

Interestingly, even though patients with *BRCA*mut disease showed more extrapelvic tumor involvement in pOC, surgical effort to achieve maximum reduction of tumor mass did not differ in both groups, at either the primary or recurrent stage. Additionally, patients with *BRCA*mut disease showed higher rates of complete cytoreduction at all times. Although the difference in patients with pOC disease with complete tumor resection did not reach statistical significance compared with the *BRCA*wt group, optimal debulking was significantly more often achieved during rOC surgery in the *BRCA*mut cohort. In a recently published study by Ataseven et al., these findings were largely confirmed in a great population of 1221 patients with pOC disease.^26^ Similarly to our findings, the *BRCA*mut cohort showed higher rates of complete tumor resection compared with their *BRCA*wt counterparts but did not reach statistical significance, and surgical complexity was not significantly different between both groups. However, median follow-up was considerably shorter, at 31 months, and the study focused only on treatment in the pOC situation. To conclude, it is conceivable that, owing to biologic characteristics of *BRCA*mut OC cells, primary occurrence of disease is earlier, but tumor invasion at recurrence seems to be less aggressive. Therefore, surgery followed by an adequate adjuvant treatment line is still very effective, even during rOC, and should be the treatment modality pursued.

As already described in previous studies, we confirmed significantly younger age at primary diagnosis in patients with *BRCA*mut disease.^27,28^ Moreover, the *BRCA*mut cohort showed significantly prolonged OS, which is also consistent with existing published literature.^29,30^ However, it has been a topic of international debate whether improved survival, operability, and response rates to therapy among the *BRCA*mut group are solely caused by the fact that these patients are younger, or whether other tumor characteristics play an important role in that process as well.

Multivariate analysis to predict complete macroscopic tumor resection in our pOC cohort revealed that only age at diagnosis had a significant effect. This finding is in line with data by Hyman et al., where patient’s age was found to be a significant predictor for surgical outcome at primary debulking, whereas *BRCA* mutation status was not.^31^ The impact of *BRCA* status on surgical outcome in rOC, however, has not been sufficiently investigated. In this study, multivariate analysis detected that *BRCA* mutation status is the only significant predictive factor for complete resection in rOC. This is in line with Estati et al., who also validated *BRCA*mut status to significantly promote complete macroscopic resection in recurrence.^32^ The rate of complete secondary resection in their study was 89.5% in the *BRCA*mut group and 65.2% in the *BRCA*wt group.^32^ This indicates that especially patients with *BRCA*mut rOC disease show altered tumor biology and consecutively treatment response that cannot just be explained by younger age. Therefore, we suggest that *BRCA* mutation status should be taken into account as a parameter when selecting patients eligible for secondary debulking surgery aiming for complete macroscopic resection.

Surgery in patients with first rOC disease has been proven to significantly prolong PFS and OS in the DESKTOP III trial only if macroscopic tumor clearance could be achieved.^3^ If there was still residual tumor mass left, patients showed worse survival compared with those treated with chemotherapy alone. Two further prospective randomized clinical trials confirmed better PFS rates in patients with rOC disease if complete macroscopic tumor resection could be achieved, but the GOG trial failed to show a significant OS benefit in patients from the surgery cohort.^33,34^ One crucial difference between the GOG trial and the DESKTOP III and SOC-1 trials is the lack of a scoring system to select patients who will more likely benefit from secondary tumor debulking. These findings underline that reliable criteria to assess eligibility of individual patients for surgical treatment are indispensable. Implementation of the AGO criteria into this decision-making process has already been proven to be a very helpful tool in clinical practice, although only complete resection rates of 67–75.5% of patients with rOC and AGO positive score were reported in recent studies.^3,4^ Conversely, complete macroscopic resection was still achievable in 48.5% patients with an AGO negative score.^4^ Hence, identifying new selection criteria for secondary tumor debulking is mandatory, especially in borderline decision cases to identify those patients in whom maximal rOC surgery is feasible without harming those patients who would not benefit from it. In this context, we suggest implementing *BRCA*mut status as a favorable factor for surgical therapy in patients with rOC disease. To our understanding, the combination of effective cytoreductive surgery with chemotherapy followed by effective maintenance therapy such as PARP inhibitors can be a key factor to achieve prolonged survival in a larger cohort of patients with *BRCA*mut OC disease. The superiority of this treatment combination has already been proven in a recent study by Marchetti et al.^35^ Additional factors, such as further homologous recombination deficiencies, need to be investigated in future studies to estimate predictive value for complete macroscopic tumor resection during primary and recurrent OC.

In our study cohort, we confirmed *BRCA*mut status to be a predictive factor for patient survival. Although patients with *BRCA*mut disease showed longer PFS at all times, a significant difference could only be measured during recurrence. Additionally, patients with *BRCA*mut disease showed a median OS benefit of 24.3 months compared with the *BRCA*wt group. These findings are partially in line with the clinical trial conducted by Bookman et al., which investigated the impact of platinum-free interval and *BRCA* mutation status on treatment and survival of patients with rOC. One major finding was that patients with *BRCA*mut disease showed a prolonged OS without reaching statistical significance.^23^ However, it has to be stated that, in the mentioned study, median follow-up was 25 months and, therefore, the observation period was possibly not long enough to detect long-term effects of *BRCA* mutation status on survival. In our study, median follow-up after first-line treatment was considerably longer, at 51.7 months, and effects of *BRCA* positivity on OS were significant. Another trial by Jorge et al. had a similar median follow-up of 49.3 months and compared survival of patients with *BRCA1* and *BRCA2* mutated OC disease treated with surgery and platinum-based chemotherapy. They calculated a median OS of 76.2 months from diagnosis for patients with positive *BRCA1* and 82.0 months for patients with positive *BRCA2* disease, which is in line with our finding of 80.6 months for the *BRCA*mut cohort.^24^

### Strengths and Limitations

The current study has some strengths and limitations. Its strengths included the multicenter trial design, which allowed gathering a large, demographically heterogeneous study cohort. Moreover, this is the first trial to focus on surgical outcome and survival of patients with *BRCA*mut disease from pOC through several courses of recurrence. Limitations include the retrospective nature of our investigation, as well as the fact that none of the *BRCA*mut patients incorporated into this study had received PARP inhibitors in their maintenance therapy regimens. Lastly, the fact that 27.4% of our study cohort were yet untested for *BRCA* mutations at the end of follow-up needs to be mentioned as a limitation. However, we deliberately decided to incorporate these patients into the *BRCA*wt cohort since our main intention was to identify effects of *BRCA*mut status. Therefore, it can be assumed that our findings definitely do not overestimate the real effect of *BRCA* mutations, since a small number of unexamined patients could also be carriers of the mutation yet were included in the negative group.

## Conclusions

Our study shows that patients with *BRCA*mut disease benefit from surgical treatment, especially in rOC, suggesting the inclusion of *BRCA* mutation status information in the decision-making process in case of recurrence. We will prospectively evaluate these findings in ENGOT-ov47-TR /NOGGO-AGO TR2/HELPER, a study that enrolls 500 patients with first relapsed ovarian cancer who are going to receive surgery followed by systemic treatment, versus systemic treatment alone on the basis of physician choice.
